# Lessons from the Bone Chapter of the Malaysian Aging Men Study

**DOI:** 10.3390/ijerph13060531

**Published:** 2016-05-25

**Authors:** Kok-Yong Chin, Wan Zurinah Wan Ngah, Soelaiman Ima-Nirwana

**Affiliations:** 1 Department of Pharmacology, Faculty of Medicine, Universiti Kebangsaan Malaysia, Kuala Lumpur 56000, Malaysia; chinkokyong@ppukm.ukm.edu.my; 2 Department of Biochemistry, Faculty of Medicine, Universiti Kebangsaan Malaysia, Kuala Lumpur 56000, Malaysia; wwanzurinah@yahoo.com

**Keywords:** bone, body mass index, osteopenia, osteoporosis, testosterone, thyroids, vitamin D

## Abstract

Male osteoporosis in Malaysia is a largely neglected problem. Therefore, a bone health study in men using quantitative ultrasonometry was launched as part of the Malaysian Aging Men Study in 2009–2012. This review aimed to summarize the findings of the aforementioned bone health study. The study examined the bone health of Chinese and Malaysian men aged 20 years and above living in Kuala Lumpur using a quantitative ultrasound device. Participants answered a questionnaire on their demographic details and physical activity status. Body anthropometry of the participants was measured and their blood collected for biochemical analysis. Results showed that a significant proportion of the Malaysian Chinese and Malay men had suboptimal bone health indicated by calcaneal speed of sound and vitamin D status. Age-related decline of the calcaneal speed of sound in these men was gradual and biphasic without ethnic difference. Body anthropometry such as height, weight, body mass index, and body fat percentage contributed to the variation of the calcaneal speed of sound in Malaysian men. Age-related changes in testosterone, insulin-like growth factor 1, and thyroid stimulating hormone also influenced the calcaneal speed of sound in these men. This study serves as a reminder that male osteoporosis in Malaysia should be an issue of concern. It is also a basis for a more comprehensive study on bone health in men in the future.

## 1. Introduction

Osteoporosis, a metabolic disease of the skeletal system, marked by decreased bone mass and degenerative changes in bone microarchitecture, is the most important cause of bone fragility and fractures in the elderly [1]. Fracture risk in the Asian population is lower compared to the Western population [2]. However, with the rapid expansion of the elderly population in Asia, the incidence of osteoporosis is projected to increase significantly. A representative Japanese study in the Tottori prefecture revealed a 3.3-fold increase in hip fractures for women and 2.3-fold increase for men aged 85 years or above, concurrent with a 3.18-fold increase of lifespan in the population over a period of 20 years (1986–2006) [3]. It is projected that 45% of the osteoporotic hip fractures will occur in Asia by 2050, an increase from 26% in 1990 [4]. Osteoporotic fractures incur significant healthcare and economic burdens. A recent study in the South Korean population indicated that the societal cost of osteoporotic fractures, inclusive of direct and indirect medical costs, increased from USD $8.8 million in 2007 to $149.3 million in 2011 [5]. Another study in the Eastern Saudi Arabian population showed that the treatment cost of osteoporotic fractures of the femur for the first year was USD $628.95 million, with a projected lifetime cost of $9.34 billion in 2025 [6]. The cost estimation of osteoporotic fractures in Malaysia is current unavailable.

Osteoporosis affects both men and women. A worldwide study of hip fracture incidence in 2000 estimated that 39% of all osteoporotic fractures occurred in men [7]. Despite a higher prevalence in women, multiple studies have highlighted that men suffer from higher morbidity and mortality after an osteoporotic fracture [8,9,10]. Pooled data from three longitudinal studies in the United States showed that the six-month mortality rate in men after a hip fracture was 19% in comparison to 9% in women [8]. In a separate single-center study conducted in Houston, USA, the 12-month mortality rate was 32% for male (mean age 80 years) and 17% female hip fracture patients (mean age 81 years) [11]. In the same study, only 4.5% of the male fracture patients received any kinds of osteoporotic treatment upon discharge, which was a sharp difference compared to 27% in female patients [11]. Male fracture patients (11%) also had fewer chances of receiving a bone mineral density (BMD) scanning upon follow up compared to female patients (27%) [11]. This highlights that male osteoporotic patients are underdiagnosed, undertreated and suffering from a lack of monitoring. There is a paucity of similar studies in the Asian region.

Osteoporosis research in Malaysia is very limited. The most comprehensive survey in hip fracture incidence was performed in 1996–1997, whereby Lee and Khir demonstrated that the rate was 218/100,000 for women and 88/100,000 for men [12]. Significant ethnic differences in hip fracture incidence were found, in which it was the highest in the Chinese and lowest in the Indians, for both sexes [12]. Data on the prevalence of osteoporosis, determined based on bone mineral density (BMD) using a dual-energy X-ray absorptiometry (DEXA) technique, was only available in women [13,14,15]. A study by Lim *et al.* in middle-aged women (mean age 51 years) living in Kuala Lumpur revealed that 8.6% of them were osteoporotic based on BMD of the spine and 21.4% based on BMD of the hip [14]. Osteoporosis research in Malaysia may have been retarded by the limited availability of DEXA devices in the country (two per million citizens) [16]. Furthermore, the DEXA machine is immobile, emits ionizing energy and requires well-trained technicians to handle [17]. A quantitative ultrasound (QUS) device has been used as an alternative in the screening of osteoporosis in Malaysia [17]. Using a QUS device, Hasnah *et al.* showed that 6% of the post-menopausal women living in a low-cost housing area in Cheras, Kuala Lumpur were osteoporotic [18]. Chan *et al.* showed that 55% of post-menopausal women visited a teaching hospital in Kuala Lumpur had suboptimal bone health based on QUS readings [19]. However, no studies have been conducted on the bone health status in men. 

Taking these knowledge gaps into consideration, bone health in Malaysian men was established as an important component of the Malaysian Aging Men Study (MAMS). The bone chapter in MAMS aimed to determine the bone health of Chinese and Malay men aged 20 years and above living in Kuala Lumpur using a calcaneal ultrasonometer. The associations between bone health indicated by calcaneal speed of sound and demographic, body anthropometric, physical activity-associated, and endocrinological factors in Malaysian men were examined. This review aimed to provide a concise summary of the findings in the bone chapter of MAMS.

## 2. Study Design

The MAMS was a cross-sectional study performed between September 2009 and December 2012. Subjects recruited were Chinese and Malay men aged 20 years and above living in Kuala Lumpur, Malaysia and its environs. They were solicited via advertisements in national newspapers, radio broadcasts, flyers, and announcements at community centers and religious venues. Inclusion and exclusion criteria of the study were stated clearly in the advertisements. Subjects with pre-existing medical conditions affecting their bone health, such as osteogenesis imperfecta, rickets, hypogonadism, hyper/hypocalcaemia, and hyper/hypoparathyroidism were excluded. Subjects receiving anabolic steroids, osteoporosis treatment, thiazides, diuretics, glucocorticoids, anticonvulsants, and lithium were also omitted. Subjects included could walk without assistance and did not suffer any fractures six months prior to the screening session. Qualified physicians performed physical examinations and medical histories of the subjects during the screening session. The study was explained to the subjects thoroughly before they gave written informed consent to participate. The Universiti Kebangsaan Malaysia Medical Centre Research Ethics Committee reviewed and approved the study protocol (Code: UKM-AP-TKP-09-2009).

Subjects answered a basic self-administered demographic questionnaire. Physical activity status of the subjects was assessed using the International Physical Activity Questionnaire (IPAQ). Body anthropometry of the subjects, including body weight, body mass index, and body fat percentage, was measured using a body composition analyzer. Bone health of the subjects was measured using a calcaneal quantitative ultrasonometer as per the recommendation of International Society of Clinical Densitometry [20]. The index of bone health generated by the machine was speed of sound (SOS). The average of three SOS readings was used for analysis to ensure consistency. The short-term *in vivo* coefficient of variation for this device was 2.9%. Fasting blood of the subjects was collected in the morning on the day of the screening session. Testosterone, estradiol, free triiodothyronine (T3), free thyroxine (T4), and thyroid stimulating hormone (TSH) were tested using an automated competitive immunoassay analyzer with direct chemiluminescence. Calcium and phosphate levels were tested using an automated colorimetric analyzer. Intact parathyroid hormone (PTH), insulin-like growth factor-1 (IGF1), 25-dihydroxyvitamin D (25(OH)D), and sex hormone-binding globulin (SHBG) were tested using enzyme-linked immunosorbent assay kits. Free and bioavailable testosterone and estradiol levels were calculated based on Sodergard’s formula [21].

## 3. The Prevalence of Suboptimal Bone Health Indicated by Calcaneal SOS in Malaysian Men

A quantitative ultrasound device is not a diagnostic tool for osteoporosis. Thus, diagnostic criteria established by the World Health Organization (WHO) based on DEXA might not be suitable for QUS [22]. Despite this, Kishimoto *et al.* had established the T-score equivalent for the QUS device used in this study, CM series, which was comparable to T-scores generated by DEXA [23]. According to their studies, a patient with a SOS T-score of −1.12 or greater was considered as normal; a SOS T-score between −1.80 and −1.12 was considered as having osteopenia; and a SOS T-score of −1.80 or lower was considered as having osteoporosis [23]. This set of cut-off values was specific to QUS device of the CM series; thus, it is not applicable to other devices.

Based on the reference values provided by the manufacturer, 3.7% of the 818 subjects screened were osteoporotic and 19.9% were osteopenic [24]. However, bone density values differ significantly by populations and the reference values used in the CM series were established in the Japanese population. Therefore, we compared the SOS values of the study population with the reference values provided by the manufacturer according to age groups (10 year intervals). Mean SOS values of the local population were found to be significantly higher compared to the Japanese reference, particularly after the age of 40 years [24]. Thus, we recalculated the T-score based on local reference value (mean SOS values of young men aged 20–29 years) and the prevalence of osteoporosis changed to 2.6% and osteopenia 21.3% [24].

The prevalence of osteoporosis generated using QUS in the current study was comparable to other studies using DEXA. A systematic review of 91 studies on osteoporosis in 51,906 Chinese men aged 20 years and above showed that the prevalence of osteoporosis was 3.2% based on lumbar spine BMD and 5.3% based on femoral neck BMD [25]. The prevalence of osteoporosis for Chinese men in Hong Kong aged 50 years and above was 6% based on hip BMD and 7% based on lumbar spine BMD. In Taiwan, the prevalence of male osteoporosis was 1.6% according to national health insurance records between 1999 and 2001 [26,27]. A survey in Thailand revealed that the prevalence of osteoporosis in men aged 20 years and above was 12.6% based on lumbar spine BMD and 4.6% based on femoral neck BMD [28].

## 4. The Relationship between Age and Calcaneal SOS

**Figure 1:**
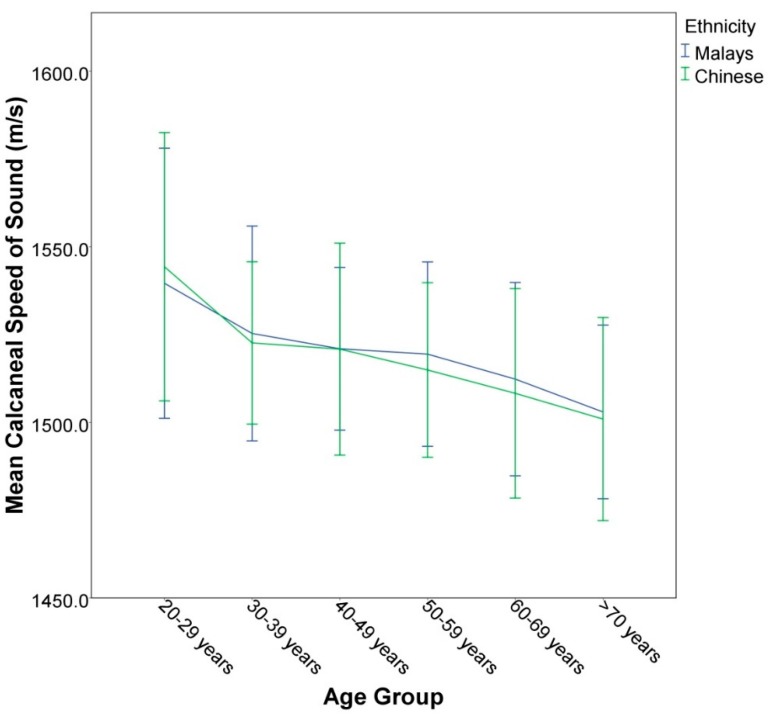
The age-trend of SOS between Chinese and Malay men in Malaysia.

Calcaneal SOS of Malaysian men showed a gradual but significant decline starting at the age of 30 years, followed by a steeper decline after the age of 60 years (Figure 1). The age trend of SOS in this study was similar to the findings of Liu *et al.* in mainland Chinese men, characterized by a drastic drop in SOS values from the 20–29 year age group to the 30–39 year age group. This decrease was also the most significant decrease in SOS per decade in their study [29]. The deterioration of bone heath with age was reflected in the exponential increase in hip fracture incidence in Malaysian men in their later life [12]. The exact reason for a steep decrease of bone health in men in their thirties remained unclear at this moment. A study by Riggs *et al.* assessing bone health in men using peripheral quantitative computed tomography (pQCT) suggested the biphasic age-related bone loss in men was due to trabecular bone loss that began before midlife, and cortical bone loss that occurred after midlife [30]. Similar age-related patterns of bone loss were not observed in other studies. A large bone health study performed in seven Asian countries using quantitative ultrasound revealed a gradual and uniform decline of the stiffness index value with the age in men [31].

## 5. The Relationship between Ethnicity and Calcaneal SOS

Ethnic difference in bone health was observed in the United States. The bone mineral content (BMC) and BMD values determined using DEXA were the highest in African Americans, followed by Hispanics and non-Hispanic Caucasians according to the third National Health and Nutrition Survey [32]. The Baltimore Men’s Osteoporosis Study also indicated that the age-related decline in femoral BMD, total hip bone mineral apparent density (BMAD), and prevalence of vertebral fractures were lower in African Americans compared to their White counterparts [33,34]. In Malaysia and Singapore, the incidence of hip fractures in men aged 50 years and above was the highest among the Chinese, followed by the Indians and the Malays [12,35]. In contrast, our study showed that the SOS value was lower in Chinese compared to Malay men, but it was not statistically significant [24] (Figure 1). Thus, variation in SOS value alone did not explain the higher fracture rate among the Chinese population in Malaysia.

## 6. The Relationship between Body Anthropometry and Calcaneal SOS

Body weight, height, bone mass index (BMI), and body fat percentage were associated with the SOS values in the subjects of this study even after adjustment for age [36]. After classifying subjects into their respective BMI categories [37], we observed that obese subjects (BMI > 30 kg·m^−2^) had significantly higher SOS value compared to subjects with normal BMI (18.5 kg·m^−2^ ≤ BMI < 25 kg·m^−2^) and overweight subjects (25 kg·m^−2^ ≤ BMI < 30 kg·m^−2^) [36]. Based on waist circumference categories [38], there was no significant difference in SOS values between subjects with (≥90 cm) and without central obesity (<90 cm) [36]. There was also no significant difference in SOS values between subjects in different classes of body fat percentages measured using bioelectrical impedance method [36]. Interestingly, stepwise regression analysis revealed that height, weight, and body fat were significant predictors of SOS in Malaysian men after adjustment for age and physical activity status [36]. Calcaneal SOS value was negatively associated with height and body fat percentage, and positively associated with body weight in the regression model [36]. In the case of body fat percentage, our results showed that conventional method of classification might be inappropriate for the prediction of bone health status.

The relationship between body anthropometry and bone health is intriguing. The negative association between height and bone health found in this study has been observed by others. Previous epidemiological studies demonstrated that taller individuals had higher risk of fractures [39,40]. Bjørnerem *et al.*, using high-resolution pQCT, demonstrated that taller women possessed wider bones with thinner and more porous cortices [41]. This provides an explanation for the negative association between height and SOS values as observed in this study, pending validation in men. Higher body weight and BMI had been shown to protect bone health, presumably by acting as a mechanical load to stimulate an increase in bone mass [42]. Body mass index reflects body size, but it does not give an accurate depiction of obesity [43,44]. The negative relationship between body fat percentage and SOS value indicated that obesity could exert negative effects on bone. Obesity is often associated with increased circulating inflammatory cytokines due to chronic inflammation [45,46]. These cytokines suppress osteoblast differentiation and promote the formation of osteoclasts [47,48]. Increased proliferation of adipocytes may compete with the formation of osteoblasts since both of them originate from mesenchymal stem cells [49]. These adipogenic factors could lead to reduced bone formation and increased bone resorption, resulting in a net bone loss.

## 7. The Relationship between Physical Activity and Calcaneal SOS

Based on the assessment by IPAQ, subjects were classified into having low, moderate, or high physical activity level. Subjects having low physical activity level had significantly lower calcaneal SOS value compared to subjects having moderate or high physical activity level [36]. Previous studies demonstrated that physical activity was associated with improved BMD assessed by DEXA and bone strength by pQCT in men [50,51]. In a study by Blanchet *et al.*, leisure physical activity was associated with calcaneal QUS indices but not BMD of the lumbar spine in a group of post-menopausal women [52].

The calcaneus is the weight-bearing bone experiencing maximal ground reaction force during physical activity [53]. Significant adaption would be expected from the skeletal deformation and straining at this site, which serves as osteogenic stimuli promoting bone growth. Bone is more responsive to weight-bearing physical activity [54]. However, the use of IPAQ represented a limitation in the current study because it did not differentiate between weight-bearing and non-weight-bearing physical activity.

## 8. The Relationship between Sex Hormones and Calcaneal SOS

Men do not experience a rapid decline of sex hormone similar to women in their midlife. The decline of testosterone is gradual and clinical testosterone deficiency is not universal in elderly men [55]. The decline of testosterone is contributed by two mechanisms. Firstly, the capacity of Leydig’s cells in the testes to synthesize testosterone in response to luteinizing hormone decreases with age [56,57]. Secondly, the increased SHBG production by the liver with age significantly limits the bioavailability of testosterone [58]. The combined effects of these two factors lead to age-related testosterone deficiency syndrome and imposes negative impacts on bone health.

Testosterone exerts direct and indirect effects on the bone. Bone cells are equipped with androgen receptors to respond directly to androgenic stimulation. Testosterone can also be aromatized in the bone or by peripheral tissues to estrogen and acts on bone cells through estrogen receptors [59,60]. A study by Falahati-Nini *et al.* showed that both testosterone and estrogen contributed to bone formation in elderly men, but only estrogen contributed significantly to suppression of bone resorption [61].

**Figure 2:**
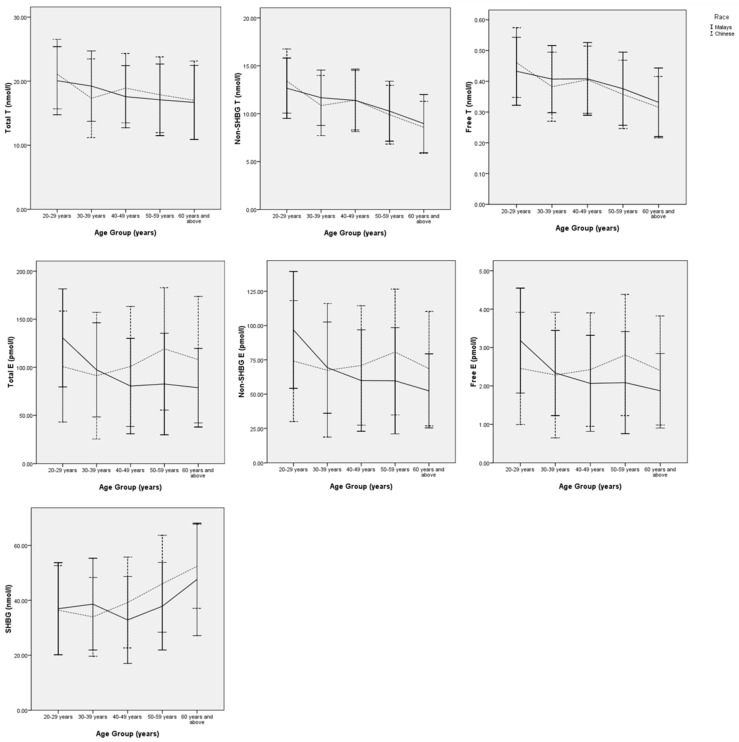
The age-trend of sex hormones between Chinese and Malay men in Malaysia [62].

Our study showed that total, bioavailable and free testosterone levels started to decline in men in their fifties. The declines of bioavailable and free testosterone were steeper compared to total testosterone. This contributed to the increase of SHBG levels, beginning in their fifties [62]. There was no specific age-trend for total, bioavailable, and free estradiol levels in Malaysian men. However, subgroup analysis based on ethnicity revealed an interesting age-related trend between Chinese and Malay men. The estradiol levels of Chinese men increased until they were in their fifties and decreased thereafter, while the estradiol levels in Malay men showed a consistent decrease with age [62] (Figure 2). The reason for this difference in trend is still unclear at this moment. Overall, there was no significant difference in sex hormone levels between Chinese and Malay men [62]. This observation was different from studies conducted in the United States. Rohrmann *et al.* demonstrated that estradiol levels were higher in African Americans compared to non-Hispanic White men, but no difference was seen in testosterone levels [31]. However, Winters *et al.* observed that testosterone levels were higher in African Americans compared to non-Hispanic White men [63].

Using multiple regression models, we showed that total, bioavailable, and free testosterone and SHBG levels were significant predictors of calcaneal SOS in Malaysian men [64,65]. Higher testosterone levels were associated with a higher SOS value in men, while higher SHBG levels predicted a lower SOS value [64,65]. No associations between SOS value and estradiol levels were found [64]. Many epidemiological studies have uncovered a positive relationship between testosterone and bone health assessed by DEXA and QUS [66,67,68,69,70]. However, there were other studies supporting that estrogen was more influential in bone health in men compared to testosterone. In the Rochester Epidemiology Study, Khosla *et al.* showed that estrogen was the only significant predictor of proximal femoral BMD after adjustment for confounders [71]. Arauja *et al.* revealed that only estradiol levels correlated with BMD at the femoral neck, hip, ultradistal radius, and spine, after adjustment for confounders but testosterone did not [72]. We postulated two reasons for the discrepancies between the current study and the previous ones. Firstly, Kuchuk *et al.* demonstrated that broadband attenuation of sound (BUA) was associated with estradiol levels in men, while SOS was associated with testosterone levels [66]. Since BUA and SOS could reflect different properties of bone [73], this indicated that estradiol could have an impact on aspects of bone quality that was undetected by SOS [60]. Secondly, phytoestrogen intake was high among the Asians [74]. This could have desensitized the bone in our subjects to the effects of estrogen.

## 9. The Relationship between Calcium Homeostasis and Calcaneal SOS

**Figure 3:**
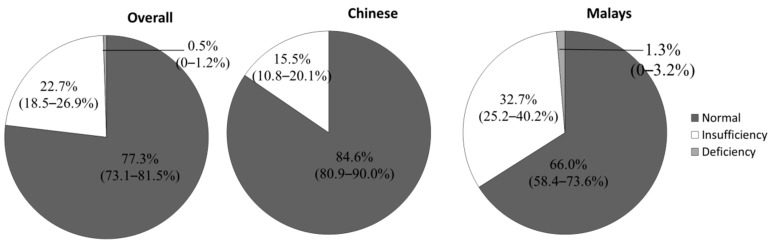
Vitamin status of Malaysian men.

The 25-hydroxyvitamin D (25(OH)D) is a prohormone synthesized cutaneously when we are exposed to ultraviolet B in the sunlight. Its deficiency leads to increased parathyroid hormone, which mobilizes the calcium reserve in our bone to the circulation, thus causing osteoporosis [75]. Vitamin D status could be divided into normal, insufficiency (30 nmol/L ≤ Vitamin D < 50 nmol/L), and deficiency (<30 nmol/L) based on the recommendation of the Institute of Medicine, USA [76]. A surprisingly large proportion of population in the tropical countries and in the sub-Sahara regions were found to possess suboptimal vitamin D level [77,78,79,80]. In our study, 22.7% of the subjects had vitamin D insufficiency and 0.5% had vitamin D deficiency [81] (Figure 3). Vitamin D insufficiency and deficiency were more prevalent among the Malay men compared to their Chinese counterparts probably due to their darker complexion [81]. Melanin competes with vitamin D synthesizing enzymes in our skin to absorb sunlight, thus hampering vitamin D production [82]. This observation was similar to a small-scale study performed in Singapore, in which 30% of the Chinese men and 48% of the Malay men had vitamin D insufficiency [83]. A study by Forrest and Stuhldreher demonstrated that vitamin D level was the lowest in African American, followed by Hispanic and non-Hispanic Whites [84]. Subjects with higher BMI had lower 25(OH)D level because vitamin D is sequestered in adipose tissue [81]. Vitamin D deficiency could also lead to metabolic syndrome, with central obesity being one of the key features [85]. Thus, the relationship between vitamin D and obesity could be two-way. The 25(OH)D level was lower in younger subjects probably due to the tendency to work indoor and the use of sunscreens [81].

There was a significant negative association between 25(OH)D and PTH level, but not serum calcium and inorganic phosphate levels in the subjects [24]. Serum 25(OH)D, PTH, calcium, and inorganic phosphate levels also did not predict SOS values in the subjects [28]. The problem of suboptimal vitamin D levels in our subjects was not serious; hence, it did not have a significant impact on their bone health. On the other hand, increased vitamin D levels might not translate to a linear increase in SOS value; thus a significant relationship was not observed.

## 10. The Relationship between Thyroid Hormones and Calcaneal SOS

The negative effects of overt hyperthyroidism on bone health were well established, but less information is available for the euthyroids [86]. Our study showed that thyroid stimulating hormone (TSH) was positively associated with SOS values in euthyroid men, but free T3 and T4 hormones were not [87]. Kim *et al.* showed that a higher TSH level was associated with a higher BMD at the lumbar spine in Korean men and a lower risk of osteoporosis in Korean women [88,89]. Similar findings were observed in the Tromso Study and the third National Health and Nutrition Examination Survey of the USA [90,91]. Receptors for TSH (TSHR) had been discovered in bone cells and TSH-influenced pathways involved in osteoclastogenesis (JNK/c-jun and NFKB) and osteoblastogenesis (LRP5, Flk-1 and Wnt5a) [92,93,94,95]. The effects of TSH on bone were independent of T3 and T4 levels, as evidenced by the severe osteoporosis found in TSHR knockout mice supplemented with thyroid hormones [93]. These provide the possible molecular mechanisms behind the effects of TSH on bone health.

## 11. The Relationship between Insulin-Like Growth Factor 1 and Calcaneal SOS

Insulin-like growth factor 1 (IGF1) is traditionally linked to regulation of growth hormone [96]. In our study, IGF1 was positively associated with SOS value in Malaysian men [87]. This finding was in accordance with several previous studies [97,98]. However, other studies found that the effects of IGF1 was more profound in women but not in men [99,100]. *In vitro* studies demonstrated that IGF1 influenced osteoblast migration, matrix synthesis, and mineralization [101,102,103]. Mice with IGF1 knockout osteoblast showed decreased bone structural, dynamic, and static histomorphometric indices [103].

## 12. Limitations

The study was cross-sectional in nature; thus, causality could not be inferred. We also used a non-randomized sampling technique; hence, generalization of the results should be performed with caution. Indians, who represent the third-largest ethnic group in Malaysia, were not recruited in the study due to logistic difficulties. However, Chinese and Malay men together represent more than 92% of the total male population in Malaysia. The QUS device used, CM-200 (Furuno Electric, Nishinomiya, Japan), only generated SOS as the bone health determinant. Another index, BUA, was not generated. Despite this, previous studies had established that SOS was closely related to BMD [104,105,106]. A meta-analysis also revealed that one standard deviation change in SOS increased the relative risk for fracture to 1.96 (CI: 1.64–2.34) [107]. The calcaneal bone was examined, but other bone segments more prone to fracture were not due to the limitation of the QUS device. This was done as per the recommendation of International Society of Clinical Densitometry [20]. Dual-energy X-ray absorptiometry was not used to assess the BMD of the subject because the device was not available to us at the time of the study. Other aspects of bone health, such as fracture risk assessments, were not performed on the subjects. Other factors influencing bone health, such as nutritional status, the use of supplements, and caffeine intake, were not taken into account in this study. Nevertheless, this study was the first study attempting to quantify the bone health status in Malaysian men and the findings were useful in planning more comprehensive studies in the future.

## 13. Conclusions

**Figure 4:**
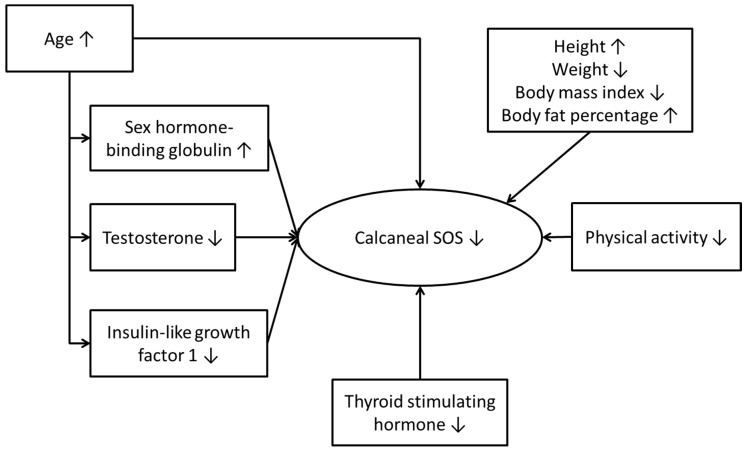
Factors influencing calcaneal SOS in Malaysian men.

A significant proportion of the Malaysian men has suboptimal bone health indicated by calcaneal SOS and vitamin D status. Age-related decline of calcaneal SOS in Malaysian men is a multifactorial problem. It is associated with changes in hormonal factors, particularly testosterone, body anthropometry, physical activity status, and other factors yet to be discovered (Figure 4). Preventive measures should be considered to retard the progress of osteoporosis in Malaysian men so that their quality of life will not be affected by this disease. These measures may be in the form of regular screening, lifestyle changes, and hormonal or nutritional supplements. However, further studies are required to determine the appropriate preventive approach.
